# Development of a system for the automated identification of herbarium specimens with high accuracy

**DOI:** 10.1038/s41598-022-11450-y

**Published:** 2022-05-16

**Authors:** Masato Shirai, Atsuko Takano, Takahide Kurosawa, Masahito Inoue, Shuichiro Tagane, Tomoya Tanimoto, Tohru Koganeyama, Hirayuki Sato, Tomohiko Terasawa, Takehito Horie, Isao Mandai, Takashi Akihiro

**Affiliations:** 1 Interdisciplinary Faculty of Science and Engineering, Shimane University, 1060 Nishikawatsu, Matsue, Shimane 690-8504 Japan; 2 Institute for Natural and Environmental Sciences, University of Hyogo/ Museum of Nature and Human Activities, Hyogo, 6 Chome, Yayoigaoka, Sanda, Hyogo 669-1546 Japan; 3 Faculty of Symbiotic Systems Science, Fukushima University, 1 Kanayagawa, Fukushima, 960-1296 Japan; 4 The Shimane Nature Museum of Mt. Sanbe, 1121-8 Tane, Sanbe-chyou, Oda-city, Shimane 694-0003 Japan; 5 The Kagoshima University Museum, Kagoshima University, 1-21-30 Korimoto, Kagoshima, 890-0065 Japan; 6 Faculty of Life and Environmental Sciences, Shimane University, 1060 Nishikawatsu, Matsue, Shimane 690-8504 Japan; 7 Alpha Hydraulic Engineering Consultants Co., Ltd., Round Cross Tsukiji 9F, 3-9-9, Tsukiji, Chuo-ku, Tokyo 104-0045 Japan; 8 T.R. Workers Co., Ltd., 1001-3-72-1 Tamagawa, Chofu-city, Tokyo 182-0025 Japan

**Keywords:** Biological techniques, Bioinformatics

## Abstract

Herbarium specimens are dried plants mounted onto paper. They are used by a limited number of researchers, such as plant taxonomists, as a source of information on morphology and distribution. Recently, digitised herbarium specimens have begun to be used in comprehensive research to address broader issues. However, some specimens have been misidentified, and if used, there is a risk of drawing incorrect conclusions. In this study, we successfully developed a system for identifying taxon names with high accuracy using an image recognition system. We developed a system with an accuracy of 96.4% using 500,554 specimen images of 2171 plant taxa (2064 species, 9 subspecies, 88 varieties, and 10 forms in 192 families) that grow in Japan. We clarified where the artificial intelligence is looking to make decisions, and which taxa is being misidentified. As the system can be applied to digitalised images worldwide, it is useful for selecting and correcting misidentified herbarium specimens.

## Introduction

Herbarium specimens were first collected about 500 years ago^1^, and approximately 380 million specimens are stored in approximately 3000 museums globally^2^. Herbarium specimens have long been used by a limited subset of researchers, such as plant taxonomists, as a reference for scientific names or voucher specimens, or as a source of information on morphology and distribution. However, research in the field of museomics^3^, in which DNA, proteins, metabolites, radioisotopes^4^, and heavy metals^5^ are extracted from specimens, has recently become prevalent. As the digitisation of label data (taxon name, collection location, collection date, images, etc.) related to plant specimens has progressed, data have been accumulated in international databases such as Global Biodiversity Information Facility (GBIF, https://www.gbif.org/) and Integrated Digitized Biocollections (iDigBio, https://www.idigbio.org/). Specimen images have become big data, and can be used freely by anybody to study, for example, the effects of climate change by examining the flowering season of certain plant species^6^. They are also beginning to be used in comprehensive research to address imminent social issues related to conservation and food security^7–9^. However, there are problems that must be resolved for the future development of these studies. One of the major problems is that the data contain misidentified specimens^10–12^. Goodwin et al.^12^ reported that at least 58% of the 4500 specimens of African gingers had a wrong name prior to a recent taxonomic study. It is difficult for non-taxonomists to notice misidentified specimens and their presence is likely to result in analyses using incorrect data. In studies that deal with big data, the amount of labour required to check for misidentifications is enormous. Misidentified specimens need to be found and corrected quickly to ensure the value of collections as a data set, but taxonomists are unevenly distributed and too few to re-examine whole specimens for their identification. There is an urgent need to develop an artificial intelligence (AI)-based plant identification system with high accuracy.

Florian Schroff et al*.*^13^ developed a system that can judge a human face. It distinguishes 8 million people from about 200 million images, and its accuracy is 99.63%^13^. The automated identification of plant species from images of a leaf^14,15^ or seedling^16–18^ is a research field with a rich recent literature, mostly concerning agriculture^19^. The LifeCLEF 2020 Plant Identification Challenge was conducted using field images of plants in addition to specimens and showed that such images can also be used for classification^20^. Recently, various researches using deep learning technology to determine species names from specimen images have been actively conducted^21,22^. In 2017, Carranza-Rojas et al.^21^ constructed a semi-automatic identification system using 113,205 images of 1000 species obtained from the iDigBio portal. GoogLeNet (InceptionV1) was used for the analysis, and the accuracy was 70.3%. In 2018, 253,733 images of 1191 species obtained from the iDigBio portal were analysed using GoogLeNet, and the accuracy was 63.0%^22^. In 2019, the Herbarium Challenge was held using 46,469 images of 683 melastome species (Melastomataceae) provided by the New York Botanical Garden, and the winning team used SeResNext-50, SeResNext-101, and ResNet-152, with an accuracy of 89.8%^2^.

In this study, we investigated the optimum number of specimens per taxa required for improving the accuracy. We also investigated whether the accuracy would improve if specimens without leaves or those with large or many holes in the leaves were excluded. In addition, we investigated which taxa were mistaken for other taxa and what part of the image was focused on when making the identification.

## Results

### Improvement of data sets and identification accuracy

**Figure 1:**
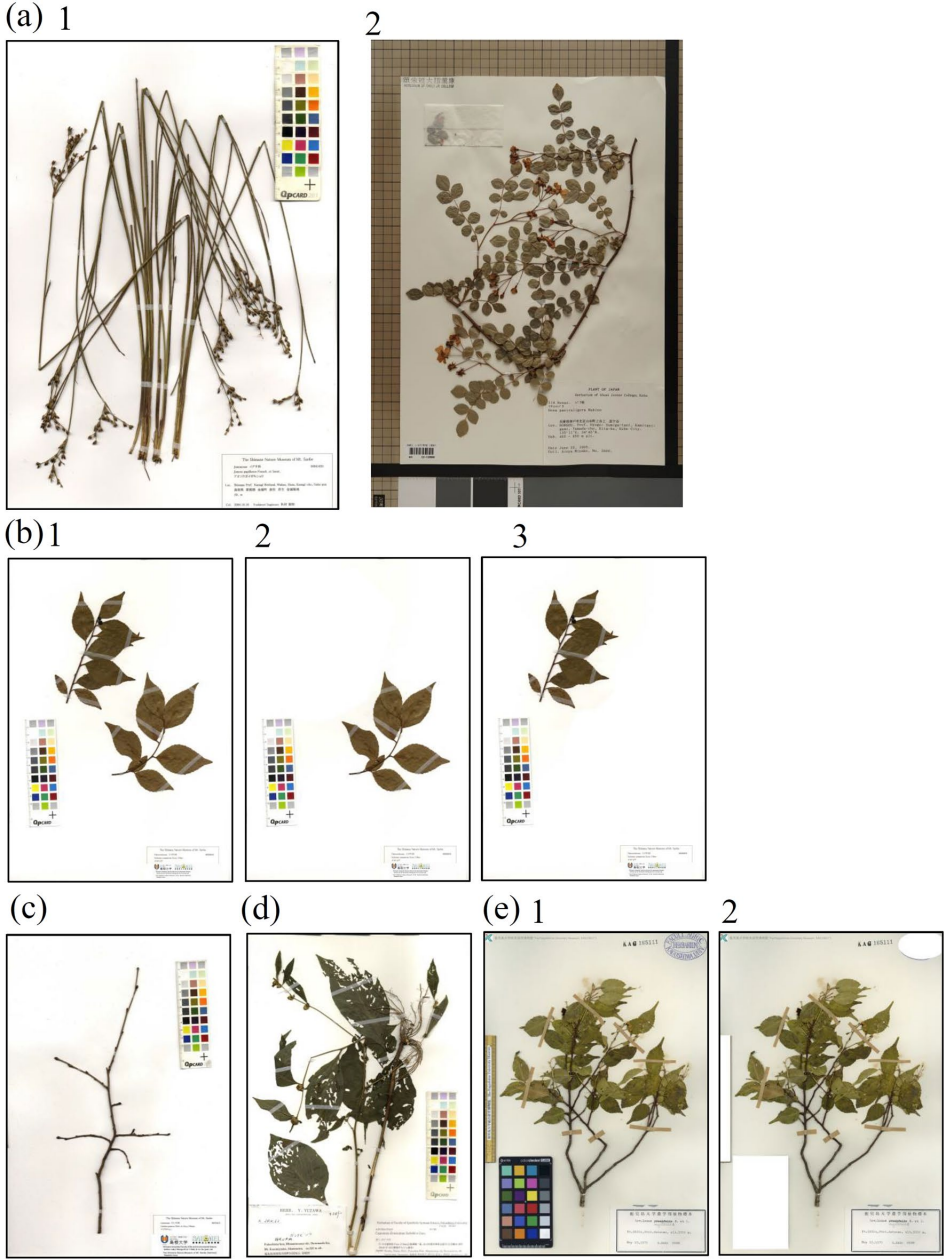
Specimen images taken with a scanner (**a**1) and with a camera (**a**2). (**b**1) Multiple individuals in one specimen image and (**b**2) image divided into two. (**c**) Specimen images showing only branches and leaves that have fallen. (**d**) Specimen image with many holes in a leaf eaten by insects. (**e**1) Specimen image with a scale, stamp, and colour bar and (**e**2) the image after the scale, stamp, and colour bar were removed.

**Figure 2:**
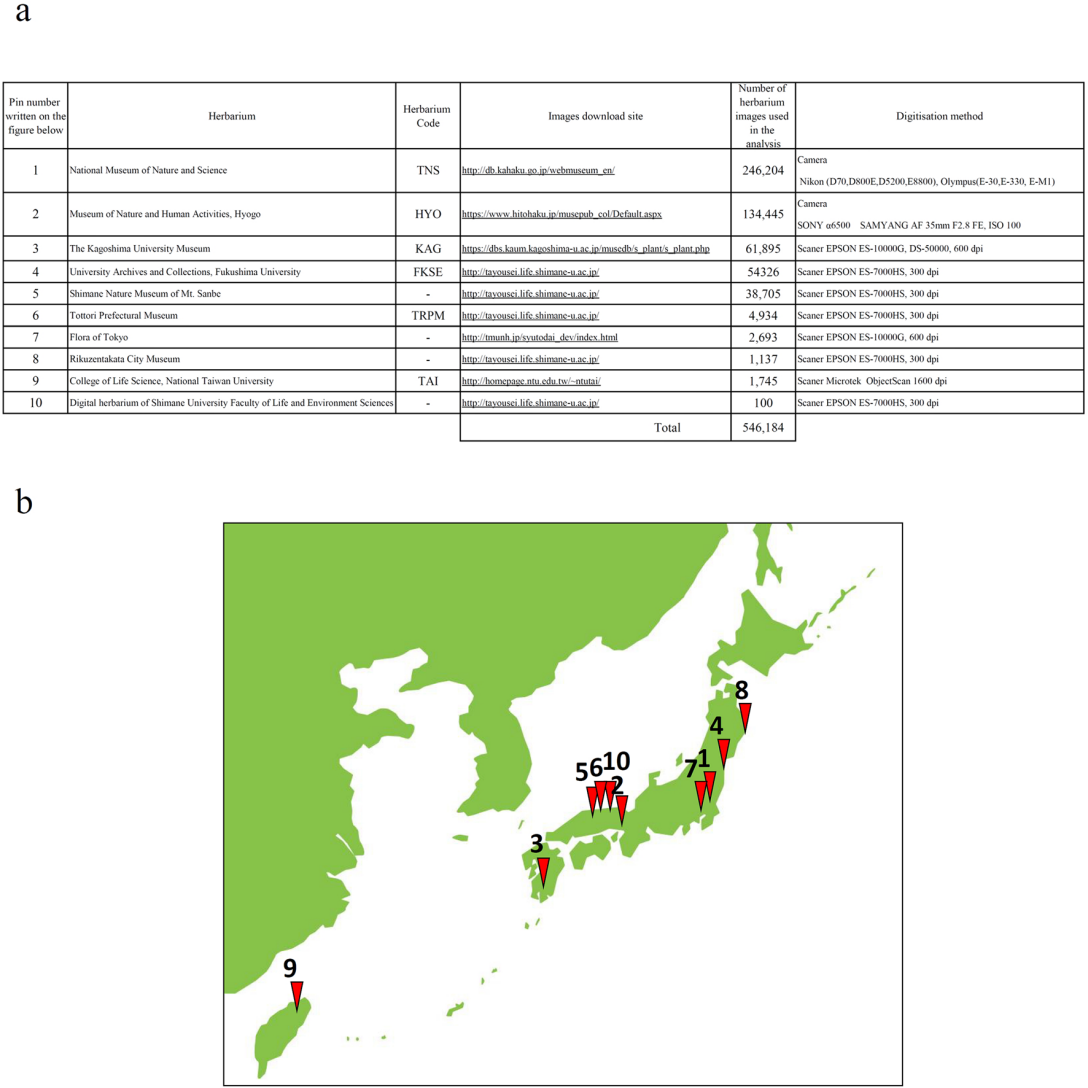
(**a**) List of herbarium-stored specimens used in this study. (**b**) Locations of specimen archives.

The targets of this study were taxa growing in Japan. In addition to images (about 290,000) digitised by the authors using a scanner (Fig. 1a1)^23^ or camera (Fig. 1a2)^24^, approximately 260,000 specimen images were downloaded from the database (Fig. 2a). Finally, 546,184 images of 3,114 taxa (2871 species, 25 subspecies, 181 varieties, 37 formas) in 219 families were obtained. Images of specimens were collected from across Japan (Fig. 2b). The number of specimens varied depending upon taxa; 838 (27%) taxa had ≤ 50 specimens, 742 (24%) had ≤ 40 specimens, 539 (17%) had ≤ 30 specimens, and 108 (3%) had ≤ 20 specimens.

Using the collected images, a plant taxa identification system was developed. For the experiments, Inception-ResNet-v2 was used, as it is the one of the most accurate function in pre-trained deep neural network^18^. The results of the first experiment showed an accuracy of 92.3% (Table 1; Supplementary Data 1). There were 319 taxa with average macro f-scores ≤ 0.6, calculated as 2 × (precision × recall)/(precision + recall), and the average number of images used in these experiments was 48 per taxa. To exclude these taxa from the analysis target in the second experiment, we decided to use only ≥ 50 images per taxa. In the case of an image containing multiple individuals or shoots in one sheet (Fig. 1b1), the individual of plants or shoots were cut out to increase the number of images (Fig. 1b2,b3). The second experiment was conducted using 534,778 images from 2,191 taxa (2,084 species, 9 subspecies, 88 varieties, and 10 formas), and the accuracy of the results increased to 93.9% (Table 1a; Supplementary Data 2).

**Table 1:** (a) List of experiments and results. (b) List of methods and results. These experiments were performed with the specimens used for the third experiment (excluding broken and misidentified specimens).

**(a)**														
Experiment No	Experiment name	Accuracy		Macro			Weighted			Number of herbarium images	Number of training images	Number of test images	Number of plant taxa	Number of family
		Top1 (%)	Top5 (%)	Recall	Precision	f-score	Recall	Precision	f-score					
1	All images	92	98	0.844	0.824	0.825	0.927	0.923	0.923	5,46,184	4,08,701	1,55,034	3114	219
2	w/o below 50 images species	94	99	0.898	0.892	0.892	0.942	0.93	0.939	5,34,778	3,85,536	1,49,242	2191	192
3	w/o broken or misidentified specimen	96	99	0.929	0.921	0.923	0.964	0.962	0.962	5,00,554	3,63,071	1,37,483	2171	192
4	w/o color-bar, stamp, scale	96	99	0.921	0.912	0.913	0.958	0.957	0.956	5,00,554	3,63,071	1,37,483	2171	192
5	Only Pteridophytes	98	100	0.946	0.947	0.945	0.985	0.984	0.984	2,04,174	1,26,218	77,956	357	32

**(b)**														
	Analysis method	Accuracy		Macro			Weighted			Number of herbarium images	Number of training images	Number of test images	Number of plant taxa	Number of family
		Top1 (%)	Top5 (%)	Recall	Precision	f-score	Recall	Precision	f-score					
	Inception-ResNet-v2	96	99	0.929	0.921	0.923	0.964	0.962	0.962	5,00,554	3,63,071	1,37,483	2171	192
	Inception-ResNet v2_base	95	99	0.913	0.905	0.906	0.955	0.953	0.953					
	Inception v3	95	99	0.909	0.901	0.902	0.953	0.951	0.951					
	VGG16	91	98	0.843	0.828	0.829	0.918	0.913	0.913					

In the second set of experiments, 11,950 images were incorrectly identified. Among them, there were 767 (6.4%) specimens that had only twigs without leaves and flowers/fruits (Fig. 1c) and/or had large or many holes in the leaves (Fig. 1d), and they were clearly misidentified. Such images were discarded, and the third set of experiments was conducted using 500,554 specimens from 2171 taxa. The accuracy in this experiment was 96.2% (Table 1a; Supplementary Data 3). In the system developed in this study, the most probable taxa were extracted as the Top-1 and the Top-5. The correct answer rate of the Top-1 was 96.2%, while the correct answer rate within the Top-5 was 99.4% (Table 1a). In this experiment, the AI misidentified 5,195 images. We investigated whether the AI had actually misidentified them, or whether this was caused by the AI correctly identifying a sample that was previously misidentified. We re-identified 181 specimens in the Herbarium of University Archives and Collections, Fukushima University (FKSE). As a result, at least 34 (19%) of the 181 specimens had been previously misidentified. We constructed the system nine times (Table 1). In the preliminary experiment, the system was constructed six times by changing the combination of training data and test data. All these results were analysed together. We selected specimens that were misidentified six times or more by the AI and re-identified them. At least 32 (28%) of 113 specimens had been previously misidentified. Subsequently, an identification system was developed focussing only on pteridophytes (353 taxa). The number of specimen images per pteridophyte taxon was higher than that of flowering plant taxa, averaging 578 per taxon (230 in the third experiment, which excluded damaged and misidentified specimens). While the number of taxa decreased to about one-sixth, the number of specimens per taxon doubled. The accuracy of the results was 98.4% (Table 1a; Supplementary Data 5). The relationship between the number of images and the average macro f-score was investigated in the analysis performed on 2171 taxa (the third experiment) and the analysis only on pteridophytes; the larger the number of images used in the analysis, the higher the average macro f-score (Supplementary Fig. 1).

Inception-ResNet v2 analysis method used in this study was compared with Inception-ResNet v2_base, Inception v3, and VGG16 using the images included in the third experiment. The method used in this study (Inception-ResNet v2) was found to be the most accurate (Table 1b). The classification accuracy of Inception-ResNet v2, Inception v3, and VGG16 showed the same tendency as classification by ImageNet, and the accuracy of Inception-ResNet v2 was the highest. The proposed method adds two 4096-dimensional, fully connected layers after the average pooling of Inception-ResNet v2. Unlike Inception-ResNet v2_base, the class of target image can be predicted from a vector with more dimensions than the number of classes; thus, the prediction accuracy is improved compared to Inception-ResNet v2 (Supplementary Data 6–8).

### The influence of collection method on identification

When collecting herbarium specimens, a collector may take several samples from the same individual plant. Even if these specimens were mounted onto different sheets, they were collected from the same plant on the same day. Therefore, these specimens may be visually much more similar than those of other samples collected from another plant of the same species, in another region, at another period of the year. Thus, the evaluation may be biased by these specimens.

Of the 2171 taxa used in this experiment, 1902 taxa (87.6%) contained samples collected on the same day. *Calamagrostis adpressiramea* Ohwi had the highest proportion of samples taken on the same day (38.7%) and an f-score of 0.888. Only two other taxa, *Polystichum* x *suginoi* Sa.Kurata (32.8%) and *Sasa megalophylla* Makino et Uchida (31.0%), exceeded 30%, and their f-scores were 0.928 and 0.692, respectively. Of the 82 taxa, which accounted for more than 10% of all samples collected on the same day and at the same location, eight were classified as woody plants (shrubs, large trees): *Eurya yaeyamensis* Masam. (f-score 0.952); *Rhododendron tashiroi* Maxim. (f-score 0.960); *Xylosma congesta* (Lour.) Merr. (f-score 0.888); *Symplocos glauca* (Thunb.) Koidz. (f-score 0.965); *Hibiscus makinoi* Jotani et H.Ohba (f-score 0.818); *Magnolia compressa* Maxim. (f-score 0.857); *Idesia polycarpa* Maxim. (f-score 0.888); and *Osmanthus marginatus* (Champ. ex Benth.) Hemsl. Of these eight taxa, there is a possibility that one individual was divided and treated as multiple specimens. The average f-score of the 82 taxa that comprised 10% of all samples collected on the same day and at the same location was 0.8992, which was lower than the average (0.958). Some of the samples used in this experiment may have been sampled from the same place on the same day, but the effect of these samples on identification accuracy was not observed.

### The influence of labels, colour bars, scales, stamps, etc. in the sample image on identification

For the Herbarium Challenge 2019 data set, the labels on herbarium sheets were removed to prevent the AI from using the plant name and other information written on the label^2^. The image input size of the training data used in this study was 299 × 299 pixels. This size was used in ImageNet^25^. It is difficult for even humans to read the written characters in these images (Supplementary Fig. 2). The effect of labels on identification was investigated using the following method. A set of 5000 correctly identified sample images were randomly selected, and the images were processed so that only the label of the sample images remained (Supplementary Fig. 2). Identification was then performed. The probability of obtaining the correct answer by chance was 0.05% (2.3 images/5000 images). Only three images were correctly identified, giving a correct answer rate of 0.06%.

In addition to the labels, some sample images contained colour bars, scales, and stamps (Fig. 1e1). To investigate the effect of these factors on identification, they were deleted from the images (Fig. 1e2) and a fourth set of experiment was conducted to investigate their effect on the identification accuracy. The accuracy was only slightly lower when using the images with the colour bars, scales, and stamps removed (Table 1a, Supplementary Data 4). From these results, it was clarified that the presence of a label, colour bar, scale, or stamp in the image does not significantly affect the accuracy of identification.

### Does AI make the same misidentifications as humans?

We investigated whether the AI could correctly identify plants that are frequently misidentified by collectors and collection managers (hereinafter referred to as experts). First, taxa that were frequently misidentified by experts were selected according to the records of identification history of the specimens in FKSE. Of the taxa stored in the FKSE that had 50 or more specimens, 17 taxa with a misidentification rate (number of misidentified or previously misidentified specimens/number of specimens) ≥ 15% were classified as ‘frequently misidentified taxa’ (Table 2). The average number of images used per taxon in the third set of experiment was 230, while the average number of images used per taxon of the 17 taxa was 328. The average macro f-score of the 2171 taxa was 0.962, while the average value of the 17 taxa was 0.890. Experts often misidentified *Platanthera tipuloides* (L.f.) Lindl. as *Platanthera minor* (Miq.) Rchb.f. In addition, *Lespedeza homoloba* Nakai was frequently misidentified as *Lespedeza cyrtobotrya* Miq. or *Lespedeza bicolor* Turcz. We investigated whether the AI also misidentified them. It was found that, for all taxa, the AI made the same mistakes as the experts (Table 2).

**Table 2:** List of 17 taxa that are frequently misidentified in the records of identification history of the specimens in the Herbarium of University Archives and Collections, Fukushima University (FKSE).

	Frequently misidentified taxa	No samples in test	Species misidentified in FKSE	No. in Top-1	No. in Top-2	Percentage in Top-1 (%)	Percentage in Top-2 (%)
1	*Platanthera tipuloides* (L.f.) Lindl.	51	*Platanthera minor* (Miq.) Rchb.f.	1	15	6	37
			*Pogonia japonica* Rchb.f.	0	1		
			*Platanthera sachalinensis* F.Schmidt	2	3		
2	*Poa nipponica* Koidz.	27	*Poa sphondylodes* Trin.	0	0	15	15
			*Poa pratensis* L. subsp. *pratensis*	1	1		
			*Poa acroleuca* Steud.	2	3		
			*Agrostis valvata* Steud.	0	0		
			*Poa annua* L.	1	0		
3	*Poa trivialis* L.	47	*Poa sphondylodes* Trin.	1	6	4	32
			*Poa pratensis* L. subsp. *pratensis*	0	0		
			*Poa acroleuca* Steud.	0	2		
			*Agrostis clavata* Trin. var. *nukabo* Ohwi	0	1		
			*Poa hisauchii* Honda	0	0		
			*Agrostis clavata* Trin. var. *clavata*	0	0		
			*Poa nipponica* Koidz	1	6		
4	*Poa hisauchii* Honda	19	*Poa acroleuca* Steud	2	1	16	47
			*Poa pratensis* L. subsp. *pratensis*	0	0		
			*Poa sphondylodes* Trin.	1	0		
			*Poa nipponica* Koidz.	0	8		
5	*Lespedeza homoloba* Nakai	178	*Lespedeza cyrtobotrya* Miq.	2	6	3	57
			*Lespedeza bicolor* Turcz.	3	52		
			*Lespedeza buergeri* Miq.	0	43		
6	*Carex leucochlora* Bunge var. *candolleana* (H.Lév. et Vaniot) Katsuy.	47	*Carex oxyandra* (Franch. et Sav.) Kudô	0	0	17	6
			*Carex leucochlora* Bunge var. *gracillima* (Akiyama) Katsuy.	5	1		
			*Carex leucochlora* Bunge var. *leucochlora*	0	1		
			*Carex discoidea* Boott var. *discoidea*	3	1		
			*Carex conica* Boott var. *conica*	0	0		
7	*Bidens pilosa* L. var. *pilosa*	13	*Bidens biternata* (Lour.) Merr. et Sherff	2	2	15	15
			*Bidens frondosa* L.	0	2		
8	*Carex otaruensis* Franch.	55	*Carex kiotensis* Franch. et Sav.	2	14	5	44
			*Carex forficula* Franch. et Sav.	1	6		
			*Carex heterolepis* Bunge	0	3		
			*Carex dimorpholepis* Steud.	0	1		
			*Carex alopecuroides* D.Don var. *chlorostachya* C.B.Clarke	0	0		
			*Carex fernaldiana* H.H.Lév. et Vaniot	0	0		
			*Carex foliosissima* F.Schmidt var. *foliosissima*	0	0		
9	*Hydrocotyle ramiflora* Maxim.	20	*Hydrocotyle maritima* Honda	1	6	5	45
			*Hydrocotyle javanica* Thunb.	0	3		
10	*Lespedeza bicolor* Turcz.	113	*Lespedeza cyrtobotrya* Miq.	3	15	13	50
			*Lespedeza homoloba* Nakai	9	40		
			*Lespedeza buergeri* Miq.	3	2		
11	*Aruncus dioicus* (Walter) Fernald var. *kamtschaticus* (Maxim.) H.Hara	51	*Astilbe odontophylla* Miq.	1	12	6	61
			*Astilbe thunbergii* (Siebold et Zucc.) Miq. var. *thunbergii*	1	18		
			*Astilbe microphylla* Knoll	0	1		
12	*Vaccinium hirtum* Thunb. var. *pubescens* (Koidz.) T.Yamaz.	99	*Vaccinium japonicum* Miq.	1	11	6	51
			*Vaccinium smallii* A.Gray var. *glabrum* Koidz.	5	39		
13	*Viola grypoceras* A.Gray var. *grypoceras*	152	*Viola kusanoana* Makino	10	26	11	31
			*Viola acuminata* Ledeb.	0	3		
			*Viola verecunda* A.Gray var. *verecunda*	2	10		
			*Viola obtusa* Makino	5	8		
14	*Lolium arundinaceum* (Schreb.) Darbysh.	43	*Poa pratensis* L. subsp. *pratensis*	0	2	2	9
			*Festuca ovina* L.	0	1		
			*Festuca rubra* L. var. *rubra*	1	1		
15	*Cardamine scutata* Thunb.	139	*Cardamine occulta* Hornem.	10	43	11	40
			*Cardamine tanakae* Franch. et Sav. ex Maxim.	5	8		
			*Nasturtium officinale* R.Br.	0	4		
			*Arabis nipponica* (Franch. et Sav.) H.Boissieu	0	0		
16	*Salix udensis* Trautv. et C.A.Mey	32	*Salix dolichostyla* Seemen subsp. *dolichostyla*	3	3	22	31
			*Salix miyabeana* Seemen subsp. *gymnolepis* (H.Lév. et Vaniot) H.Ohashi et Yonek.	2	2		
			*Salix integra* Thunb	1	0		
			*Salix triandra* L. subsp. *nipponica* (Franch. et Sav.) A.K.Skvortsov	1	5		
			*Salix futura* Seemen	0	0		
17	*Festuca rubra* L. var. *rubra*	32	*Lolium arundinaceum* (Schreb.) Darbysh.	1	0	6	3

We investigated whether the AI and experts tend to make the same misidentification. Although bluegrasses (*Poa* spp., Poaceae) are morphologically similar to each other, the AI rarely misidentified *Poa nipponica* Koidz. (Poaceae) as *Poa trivialis* L., *Poa annua* L., and *Poa pratensis* L. subsp. *pratensis*, but often as *Corydalis pallida* (Thunb.) Pers. var. *tenuis* Yatabe (Papaveraceae) and as *Pilea hamaoi* Makino (Urticaceae). Experts do not misidentify *C. pallida* var. *tenuis*, *Pilea hamaoi*, and *Poa nipponica* because they are very different in shape. Therefore, we did not understand why the AI confused these species.

**Figure 3:**
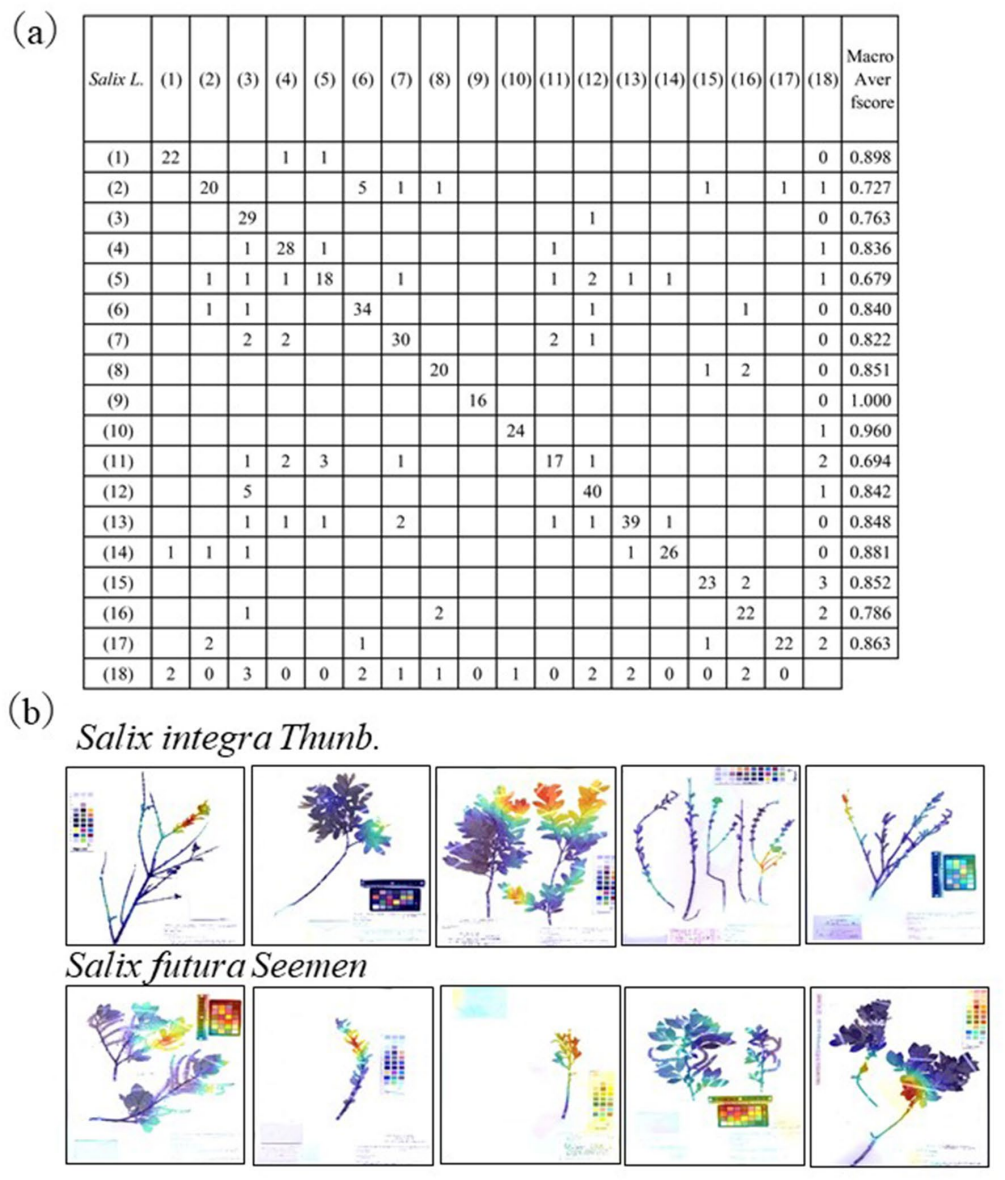
(**a**) A cross-tabulation table was created for the recall and precision values of 17 taxa of *Salix* L in the third experiment. (1) *S. integra* Thunb., (2) *S. futura* Seemen, (3) *S. pierotii* Miq., (4) *S. udensis* Trautv. et C.A.Mey., (5) *S. miyabeana* Seemen subsp. *gymnolepis* (H.Lév. et Vaniot) H.Ohashi et Yonek., (6) *S. vulpina* Andersson subsp. *vulpina*, (7) *S. dolichostyla* Seemen subsp. *serissifolia* (Kimura) H.Ohashi et H.Nakai, (8) *S. vulpina* Andersson subsp. *alopochroa* (Kimura) H.Ohashi et Yonek., (9) *S. eriocataphylla* Kimura, (10) *S. japonica* Thunb., (11) *S. dolichostyla* Seemen subsp. *dolichostyla*, (12) *S. eriocarpa* Franch. et Sav., (13) *S. triandra* L. subsp. *nipponica* (Franch. et Sav.) A.K.Skvortsov, (14) *S. gracilistyla* Miq., (15) *S. caprea* L., (16) *S. chaenomeloides* Kimura, (17) *S. sieboldiana* Blume var. *sieboldiana*, (18) others. (**b**) Results of Grad-CAM analysis of *Salix integra* and *Salix futura*. Red indicates the more important parts while blue represents less important parts.

To clarify what kind of taxa are selected for the Top-2 by AI when AI identifies taxa successfully, we selected the 1022 images that the AI identified correctly. The taxa that the AI identified as the Top-2 were checked against the corresponding images. It was found that 443 (43.3%) of the 1022 images were the taxa that are also misidentified by experts (Table 2) (Supplementary Data 3). The third case is of willows (*Salix* spp.), dioecious trees that are difficult to identify, even for experts^26^. Floral characters are important for species diagnosis, but those of willow species are small, less than 10 mm long, and each specimen contains either female or male flowers. The shape of leaves can be useful for taxon recognition, but willow leaves usually start to emerge after anthesis. All the willow specimens were used as training data without separating the males and females, and the image input size was 299 × 299 pixels, which is too small for the small floral organs to be recognized. A cross-tabulation table was created for the recall and precision values of 17 willow taxa (Fig. 3a), and it was found that willow taxa were often misidentified within the same genus. The Gradient-weighted Class Activation Mapping (Grad-CAM) results (an analysis method that displays the most important parts in different colours during AI identification^27^) showed that the AI displayed a tendency to use the inflorescences and infructescence, and some of the branches to which they were attached for identification, and then use entire leaves for identification after using the infructescence (Fig. 3b).

### Is AI misidentifying taxa of the same genus?

**Figure 4:**
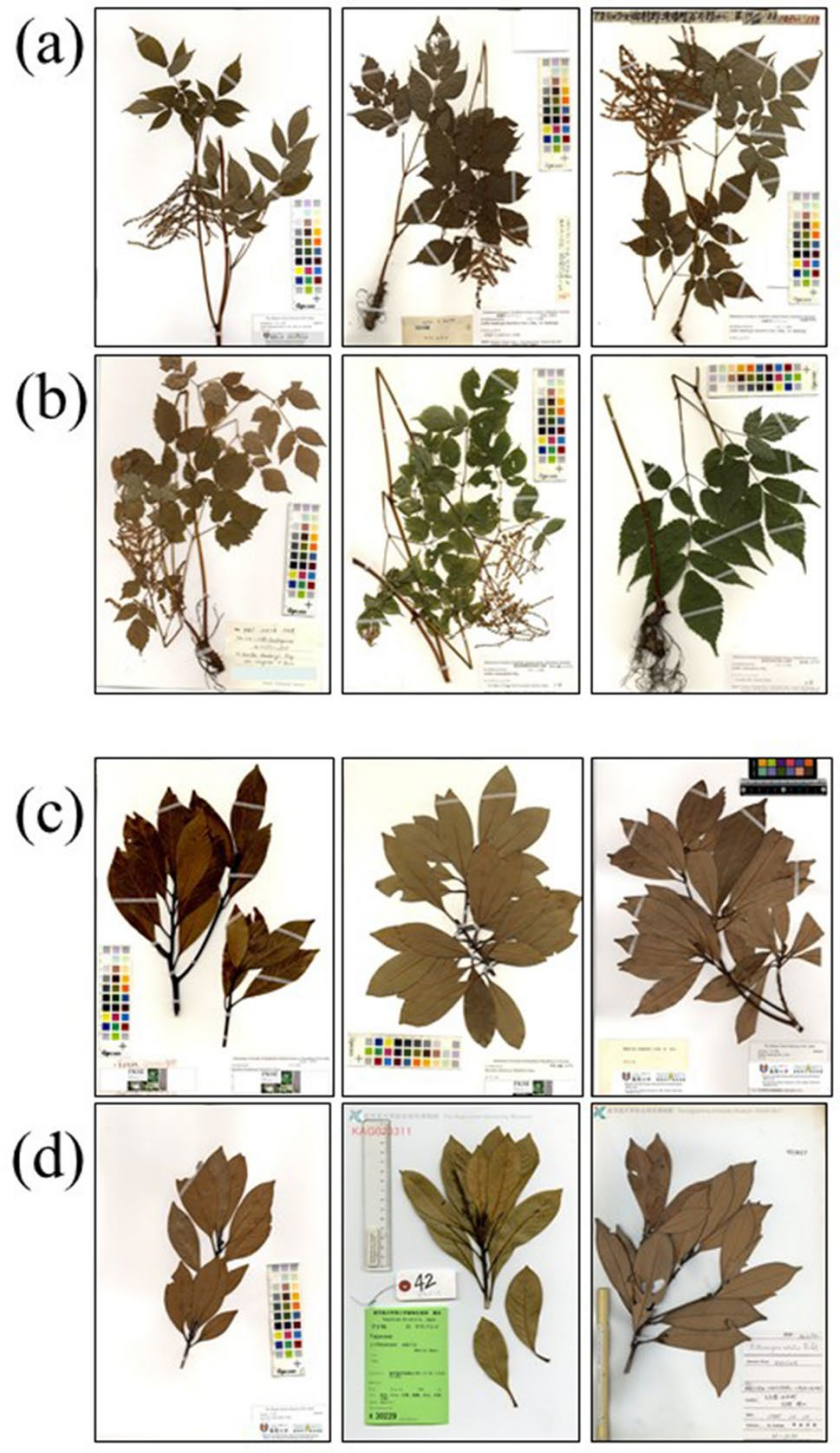
(**a**) *Astilbe thunbergii* (Siebold & Zucc.) Miq. var. *thunbergii*, (**b**) *Astilbe odontophylla* Miq., (**c**) *Machilus thunbergii* Siebold & Zucc., and (**d**) *Lithocarpus edulis* (Makino) Nakai. Species (**a**) and (**b**) and species (**c**) and (**d**) are different plants with similar morphologies. These species are often misidentified by experts and were misidentified by AI in this study.

Species misidentified by experts are mostly within the same genus. Therefore, we investigated whether the taxa misidentified by the AI belonged to the same genus. We examined the genera of the Top-5 taxa for 137,483 images that were correctly identified by the AI. All five of the Top-5 taxa were from the same genus (including the Top-1 correctly identified taxon name) in 4.6% (6293) of the cases, four in 9.5% of cases, three in 17.1% of cases, and two in 27.5% of cases. The genera of the Top-2–5 taxa differed from that of the Top-1 in 41.6% of cases. Even experts sometimes mistakenly identify *Gynostemma pentaphyllum* (Thunb.) Makino (Cucurbitaceae) as *Causonis japonica* (Thunb.) Raf. (Vitaceae), or *Aruncus sioicus* (Walter) Fernald var. *kamtschaticus* (Maxim.) H. Hara (Rosaceae) as *Astilbe thunbergii* Miq. var. *thunbergii* (Saxifragaceae), which are in different families. We investigated whether the AI misidentified these species in the same way, and the same misidentifications were found (Fig. 4).

### Identification of parts where AI is important for identification

**Figure 5:**
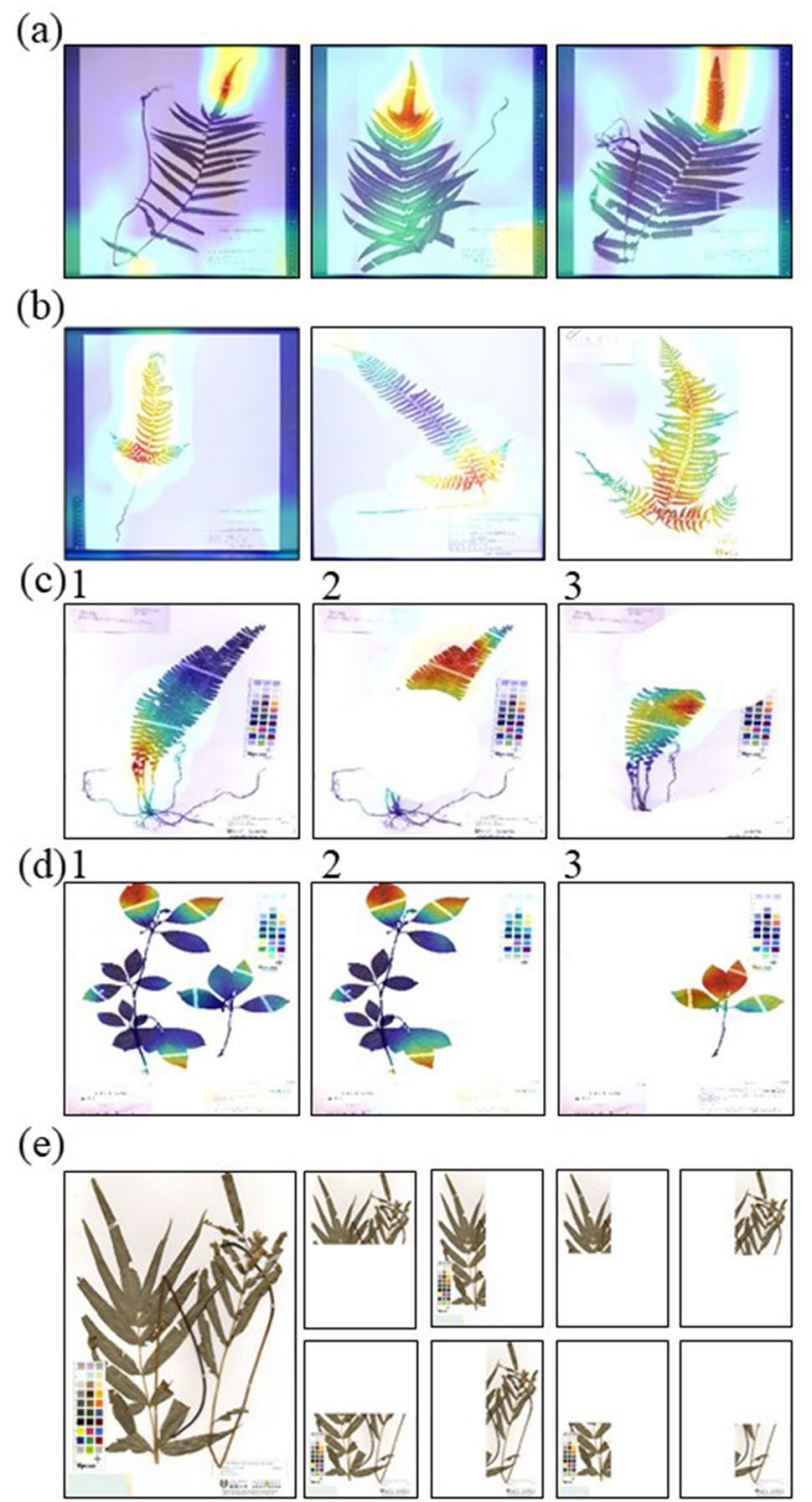
Results of Grad-CAM analysis of (**a**) *Thelypteris acuminata* (Houtt.) C.V. Morton and (**b**) *Polystichum tripteron* (Kunze) C. Presl. (**c**) In the Grad-CAM analysis, only part of the specimen was considered important (red). (**c**2) Images in which the focal part was cut out and (**c**3) images in which only the non-focal part was cut out were created and used for Grad-CAM analysis. (**d**1) Multiple individuals in one specimen image. The image was divided into two images (**d**2, 3) and used for Grad-CAM analysis. (**e**) The sample image was halved vertically and horizontally, and further divided it into four both vertically and horizontally.

Easy-to-identify pteridophytes include *Thelypteris acuminata* (Houtt.) C.V. Morton with its long terminal leaflet and *Polystichum tripteron* (Kunze) C. Presl with its long, cross-shaped basal pinnae. The average macro f-score of *T. acuminata* was 0.993, and that of *P. tripteron* was 0.998. Looking at the Grad-CAM analysis results, we found that the characteristic parts for each species were captured (Fig. 5a,b). For *T. acuminata* 352 of the 648 images (54.3%) focused on the apical part of lamina. For *P. tripteron* 1,320 of the 1,381 images (95.5%) focused on the long, cross-shaped basal pinnae.

Using Grad-CAM, we identified the parts of the image that are important for AI identification. First, we investigated the Grad-CAM analysis results and selected 287 images that were identified by focussing on a particular part of the image (Fig. 5c1). Subsequently, from each image two images were created—one in which the non-focal parts were cut out (Fig. 5c2) and the other in which the focal parts were removed (Fig. 5c3). These images were then analysed. The accuracy rate for the images containing only the non-focal parts decreased to 72.4%, while the correct answer rate for the images that included only the focal part was 54.0%. Although the area of the focal part was small and the area of the non-focal parts was large, the accuracy rate of images containing only the focal parts was low. This was the opposite of what was expected. From these results, it was expected that AI would first look at the whole and narrow down, and then look at specific parts to narrow down further. After processing an image that contained two individuals in one specimen (Fig. 5d1) to produce an image containing only one individual (Fig. 5d2 & 3), the accuracy was 82%. We prepared an image in which the sample image was halved vertically and horizontally and further divided it into four both vertically and horizontally (Fig. 5e). When tested, the accuracy decreased to 54%.

### Publication of the system

**Figure 6:**
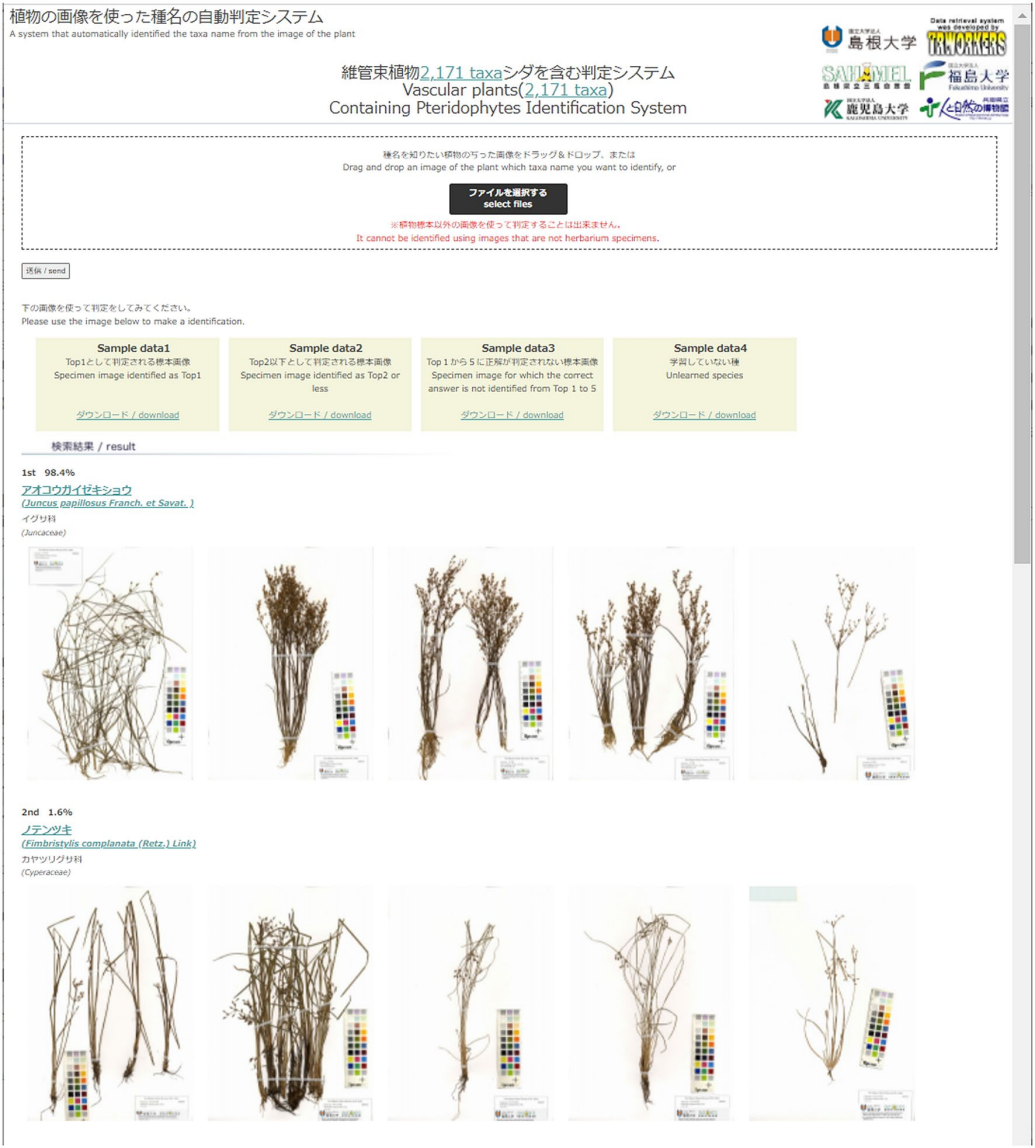
A system that automatically identified the taxa name from an image of a plant (http://tayousei.life.shimane-u.ac.jp/ai/index_all.php). The identification system for pteridophytes is located at http://tayousei.life.shimane-u.ac.jp/ai/index_Pteridophytes.php. Drag and drop an image of the plant for which you want to identify the taxa name, or select files and click the send button. Plant taxa from Top-1 to Top-5 are displayed as candidates, and the accuracy is also displayed.

The identification system we developed in this study is open on the web site for 2,171 taxa (http://tayousei.life.shimane-u.ac.jp/ai/index_all.php) and for only pteridophytes (http://tayousei.life.shimane-u.ac.jp/ai/index_Pteridophytes.php). When an image file is dragged and dropped in the web site the Top-1 to Top-5 taxa are displayed with their probability of accuracy (Fig. 6).

## Discussion

### How to improve identification accuracy?

In this study, it was clarified whether the method of selecting training data contributed to the improvement of the accuracy of the identification system. It was the deletion of poor-quality specimens and the deletion of specimens with obvious misidentification from the training data that contributed to the improvement of the accuracy of the identification. This is the first study that has been done to improve the accuracy by changing the quality and quantity of the specimen. The data sets for the Herbarium Challenge 2021 (https://www.kaggle.com/c/herbarium-2021-fgvc8/data) contained multiple images without plants, only envelopes with seeds (3482.jpg, 3727.jpg. 6990.jpg, 2191201.jpg, 857396.jpg, etc.), and no plants (1779.jpg, 572646.jpg, 485802.jpg, 103392.jpg, 1296004.jpg, etc.). By deleting such data, it was thought that the accuracy of the data would be further improved. In the experiments conducted using only images of pteridophytes, 204,174 images were used in this study. Of these, 88.2% were stored in the National Museum of Nature and Science, Tokyo (TNS). Many pteridophyte taxonomists have been involved in identification of the pteridophytes specimens at TNS during the two projects of exhaustive flora of the Japanese pteridophytes^28,29^, and the stored specimens have been databased with high identification accuracy. This was considered one of the causes of the high accuracy of the pteridophyte identification experiment. These results indicated that the accuracy can be improved by such work, which can be easily judged by the human eye. Re-identification of the misidentified specimens by AI revealed that at least 19% were misidentified. From this, it was found that in order to further improve the identification accuracy, it is necessary to improve the quality of the training data rather than improving the method.

Plants vary in size and shape depending on the stage and environment in which they grow, and some have flowers and fruits. Furthermore, different colours and morphologies are shown, depending on the method used to make the specimens^30^. To further improve the accuracy, it is necessary to increase the number of specimens per taxon with low average macro f-scores. Although there were taxa with a high average macro f-score, even if the number of specimens is approximately 60 (for example, *Asplenium setoi* N. Murak. et Seriz., *Scrophularia musashiensis* Bonati, *Stewartia monadelpha* Siebold et Zucc., and *Styrax shiraianus* Makino), there were some taxa with average macro f-scores ≤ 0.85, even when the number of specimens was ≥ 250 (for example, *Abelia spathulata* Siebold et Zucc. var. *spathulata*, *Agrostis gigantean* Roth, *Arisaema japonicum* Blume, *Carex kiotensis* Franch. et Sav., *Cirsium tonense* Nakai var. *tonense*, *Persicaria odorata* (Lour.) Soják subsp. *Conspicua* (Nakai) Yonek., *Persicaria japonica* (Meisn.) Nakai ex Ohki var. *japonica*, *Persicaria maculosa* Gray subsp. *hirticaulis* (Danser) S. Ekman et Knutsson var. *pubescens* (Makino*)* Yonek., and *Vandenboschia kalamocarpa* (Hayata) Ebihara). In the latter case, the AI often misidentified these taxa as other taxa in the same genus. In other words, they were misidentified as taxa with similar morphologies. To solve this problem, it is necessary to use more accurately identified specimens for taxa with low average macro f-scores.

The effects of specimen labels, stamps, rulers, colour bars, etc. contained in the specimens on the identification were investigated and it became clear that the accuracy of the identification did not increase even if these were removed.

### Similarities and differences between AI and human identification methods

From previous studies, it was not clear what the AI was using to make its decisions. In this study, the Grad-CAM analysis revealed the important areas in a specimen image used for AI identification. As the accuracy decreased if parts of the image were removed and only a section of the plant was used for identification, or when the image was divided and the identification made using a reduced area (Fig. 5e), the AI appeared to first observe the whole plant and then add specific characteristic parts. The identification method of the AI may be similar to that performed by experts.

It was also not apparent in previous studies which taxon is mistaken for which taxon. In this study, we created a cross-table (Fig. 3a, Supplement Data 1–8) and investigated this information. As a result, it became clear that the AI made mistakes in the taxa of the same genus. Furthermore, it became clear that taxa of different genera and families that are similar in their morphology were also mistaken as in the case of experts.

In the willow genus (*Salix* spp.), it was found that the identification method is different between AI and experts because the floral parts that experts are paying attention to are too small for the AI. If the part required for identification is small, it was thought that identification would be possible by preparing an enlarged image of the part and training it. In the case of willows, identification was possible at a certain level without using such a small part, so it was considered that the accuracy of identification could be improved by increasing the number of specimens to be trained.

### Utilization of the system created in this study

In Japan, the number of plant taxonomists who are able to classify plant taxa accurately is declining, and this trend is expected to continue. While the number of people who can correctly identify taxa is decreasing, the need for environmental investigation is increasing owing to active human activities and environmental change. Thus, it is necessary to develop technology that can help non-experts to correctly identify taxa. The identification system developed in this study is a good candidate.

By constructing a system multiple times by changing the combination of training data and test data, it is possible to select particular specimens in which AI makes a mistake in identification multiple times. Since about 28% of the specimens selected in this way were misidentified and then the correct specimen data was registered in GBIF, it can be said that our system is a good one for selecting the misidentified specimens and correcting the data. Herbaria and databases are full of misidentified specimens^10–12^. The method developed in this study is considered to be effective for the correction of such specimens and the reduction of erroneous data due to misidentified specimens in the database.

## Methods

### Digitisation of specimens and collection of digitised specimen images

Specimens in the FKSE, Tottori Prefectural Museum, Rikuzentakata City Museum, Kagoshima University Museum, Shimane Nature Museum of Mt. Sanbe, and Shimane University Faculty of Life and Environment Sciences were digitised using a scanner (EPSON DS-50000G, ES-7000HS, or ES-10000G). The method has been described previously^23^. Specimens from the Museum of Nature and Human Activities, Hyogo were digitised using a camera (SONY α6500 Samyang AF 35 mm F2.8 FE, ISO 100), as described previously^24^. Digitalised images of TNS, College of Life Science, National Taiwan University, and Flora of Tokyo specimens were downloaded from the website (the URL is shown in Fig. 1a.). The TNS specimens were digitised using a camera, while the College of Life Science, National Taiwan University, and Flora of Tokyo specimens were digitised using a scanner (Fig. 2a). The images were downsized to 299 × 299 pixels for input size in this study (Supplementary Fig. 2).

### Deep learning model

It has been clarified that deep learning, which was used in this study, is more accurate than non-deep methods^31^. A convolutional neural network is a neural network model mainly consisting of convolutional, pooling, and fully connected layers (Supplementary Fig. 3). The convolutional layer has a weight parameter called a filter. The input image is converted into a feature map by applying the filter. The pooling layer extracts representative values from a specific region and reduces the spatial size. In the fully connected layer, all the nodes in the layer are connected to each other, and each edge has an independent weight. Different convolutional neural network models can be designed depending on the composition of the layers. In recent years, models such as VGG^32^, Inception^33^, and ResNet^34^ have been confirmed to be highly accurate. Inception is composed of inception blocks that integrate the results of multiple convolutional and pooling processes within a single layer. ResNet has a shortcut connection that prevents gradient loss. Inception-ResNet-v2 consists of an inception block with an added shortcut connection, and has been shown to possess high classification accuracy^25^. The performance of this model was evaluated using the ImageNet dataset with 1000 different classes, and the Top-5 accuracy was approximately 95%. In this study, we used Inception-ResNet-v2 with two additional fully connected layers, with 4096 nodes each, after average pooling, to perform classification on a dataset with a large number of classes. The output of the first fully connected layer was normalised using Batch Normalisation. In Inception-ResNet-v2, the number of nodes after average pooling is 1792, so if the number of classes exceeds 1792, the probability of belonging to each class is predicted using fewer nodes than the number of classes. By adding a fully connected layer, it becomes possible to predict the probability of belonging to each class using a larger number of nodes.

### Evaluation

To evaluate our experiments, we examined the accuracy and f-score of taxa classification. Accuracy is defined as the rate of correct answers among all test data. Top-1 accuracy is considered correct when the class ranked first in the prediction results is the correct answer. Top-5 accuracy is considered correct when the top five classes in the prediction results contain the correct answer.

The f-score is defined as a harmonic mean of precision (*P*_*i*_) and recall (*R*_*i*_).

$$P_{i} = \frac{{a_{i} }}{{a_{i} + b_{i} }}$$

$$R_{i} = \frac{{a_{i} }}{{a_{i} + c_{i} }}$$

$$f_{i} = \frac{{2 \times P_{i} \times R_{i} }}{{P_{i} + R_{i} }}$$

In these formulae, for a class (*i*), *a*_*i*_ is the number of positive answers to positive samples; *c*_*i*_ is the number of negative answers to positive samples; and *b*_*i*_ is the number of positive answers to negative samples. The f-score of each class (*f*_*i*_) is defined as a harmonic mean of precision and recall, and the whole f-score is the macro average and weighted average.

### Method of removing stamps, colour bars, and scales from images

In-house software was developed to remove stamps, colour bars, and scales with a priori knowledge of their shapes and colours.

## Supplementary Information

Supplementary Information 1.

Supplementary Information 2.

Supplementary Information 3.

Supplementary Information 4.

Supplementary Information 5.

Supplementary Information 6.

Supplementary Information 7.

Supplementary Information 8.

Supplementary Information 9.

Supplementary Information 10.

Supplementary Information 11.

## Data Availability

Some of the data used in this study can be downloaded from the database (Fig. 2a). The processed and other data for which we have the copyright are available upon reasonable request emailed to the corresponding author. However, data for which we do not have the copyright are unavailable.
