# Supplementary figures and images for: Development of a system for the automated identification of herbarium specimens with high accuracy
Source: Sci Rep. 2022 May 16;12:8066. doi: 10.1038/s41598-022-11450-y (PMC9110755; doi:10.1038/s41598-022-11450-y)

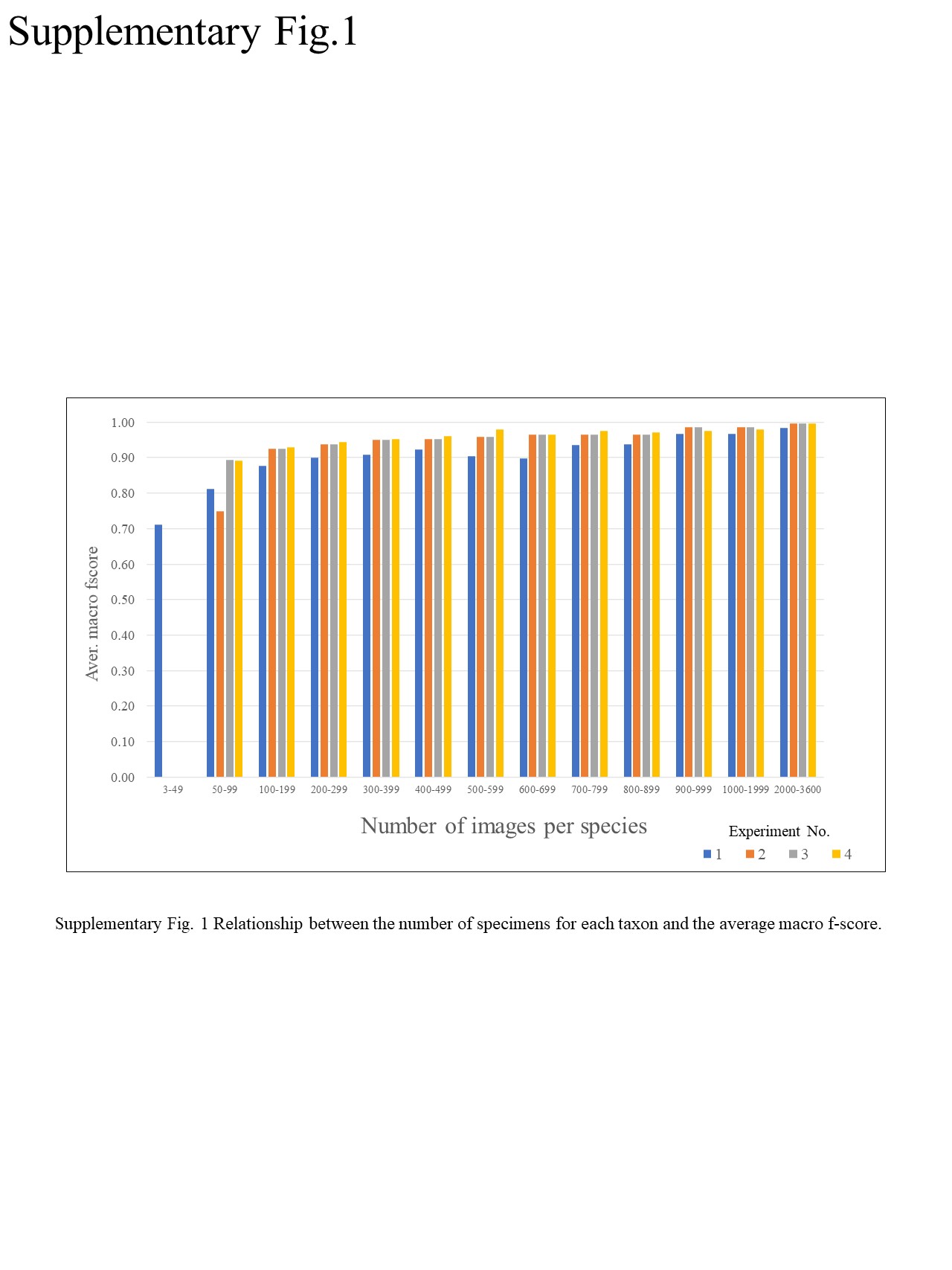

Supplement: Supplementary file 9 — Supplementary Information 9. [file 41598_2022_11450_MOESM9_ESM.jpg]

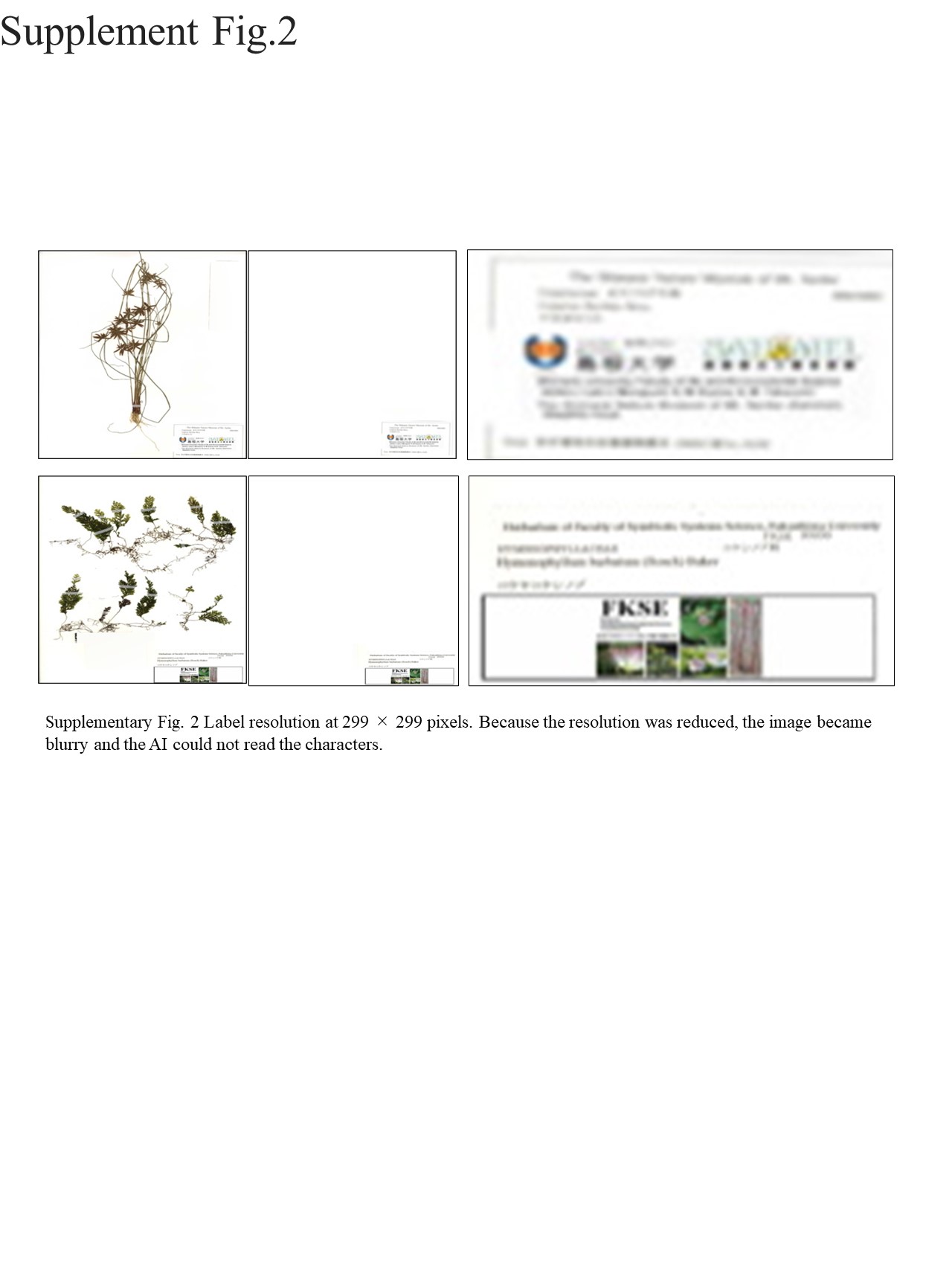

Supplement: Supplementary file 10 — Supplementary Information 10. [file 41598_2022_11450_MOESM10_ESM.jpg]

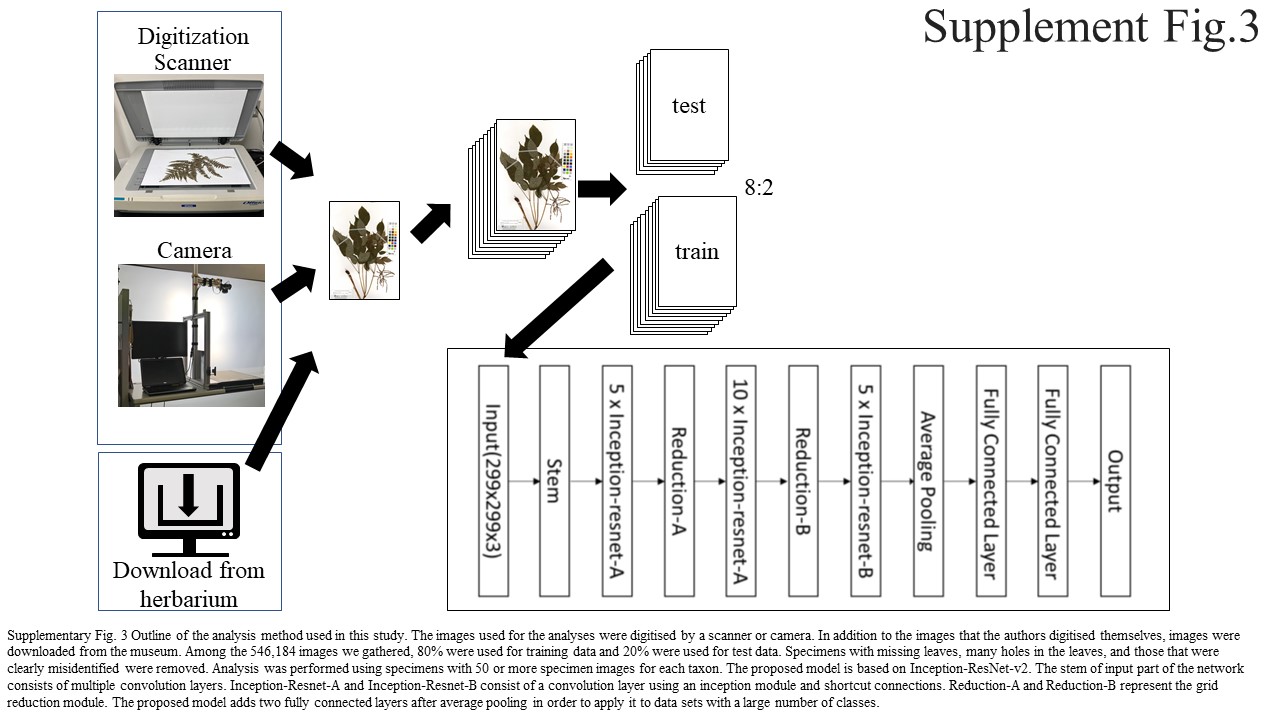

Supplement: Supplementary file 11 — Supplementary Information 11. [file 41598_2022_11450_MOESM11_ESM.jpg]
